# Nonlinear association between TyG-BMI and MAFLD: A cross-sectional study

**DOI:** 10.1371/journal.pone.0331140

**Published:** 2025-10-16

**Authors:** Qiuping Wang, Yuan Su, Jing Niu, Yan Wang, Liping Liu, Yanhong Hao

**Affiliations:** 1 Department of Ultrasound, Shanxi Children’s Hospital, Shanxi Maternal and Child Health Care Hospital, Taiyuan, Shanxi, China; 2 Department of Ultrasound, First Hospital of Shanxi Medical University, Taiyuan, Shanxi, China; 3 Department of Ultrasound, Health Team of Tai yuan Branch of Shanxi Armed Police Corps, Taiyuan, Shanxi, China; 4 Department of Interventional Ultrasound, First Hospital of Shanxi Medical University, Taiyuan, Shanxi, China; The First Affiliated Hospital of Soochow University, CHINA

## Abstract

**Background:**

The relationship between the triglyceride glucose-body mass index (TyG-BMI) and the incidence of metabolic associated fatty liver disease (MAFLD) is a trending research area. This association is highly important in the realms of healthcare and public health.

**Objective:**

To explore the impact of TyG-BMI on MAFLD and its contribution to the evolution of diverse phenotypes of fatty liver.

**Methods:**

In this cross-sectional study, curve fitting was used to assess the relationships between TyG-BMI and MAFLD as well as different MAFLD phenotypes, and multivariate ordinal logistic regression methods were employed to explore the stability of these relationships.

**Results:**

Curve fitting revealed a nonlinear relationship between TyG-BMI and MAFLD, with a critical threshold of approximately 176.78. Similar relationships were observed between TyG-BMI and different fatty liver phenotypes. Multivariate logistic regression revealed that the TyG-BMI was independently associated with different fatty liver phenotypes (nonfibrotic MAFLD odds ratio (OR) = 11.29; 95% confidence interval (CI) = 8.38–15.21, *p* < 0.001; fibrotic MAFLD OR = 37.24, 95% CI = 25.92–53.51, *P* < 0.001). Even after full variable adjustment, this relationship remained stable (nonfibrotic MAFLD OR = 5.99, 95% CI = 3.25--11.06, *P* < 0.001; fibrotic MAFLD OR = 5.24, 95% CI = 2.28--12.06, *P* < 0.001). Correlation analysis revealed a positive correlation between TyG-BMI and CAP (correlation coefficient, 0.60) and a weak positive correlation with SWE (correlation coefficient, 0.29).

**Conclusions:**

The TyG-BMI manifests a nonlinear relationship with the risk of developing distinct MAFLD phenotypes, suggesting its utility as a pivotal metric in the formulation of screening and preventive strategies for MAFLD.

## Introduction

Non-alcoholic fatty liver disease (NAFLD) is often associated with metabolic syndrome. In 2020, an international expert consensus recommended redefining NAFLD as metabolic-associated fatty liver disease (MAFLD) [1,2]. Unlike the exclusionary diagnostic criteria of NAFLD, MAFLD is primarily defined by positive diagnostic criteria that encompass hepatic steatosis and metabolic dysfunction. Compared with NAFLD: MAFLD provides a more precise description of fat deposition in the liver, accounting for both alcohol-related and systemic metabolic factors that contribute to metabolic dysregulation [3]. In addition, studies have shown that compared with NAFLD, the MAFLD definition may better identify significant fibrosis [4]. Several research have reported that individuals with MAFLD are at increased risk for numerous adverse health outcomes, including chronic kidney disease (CKD) [5], elevated cardiovascular mortality rates [6,7], and a spectrum of other liver-related morbidities [8,9]. The increasing incidence of MAFLD has significantly contributed to an increased financial burden on the societal economic framework [10].

Despite the growing prevalence and severe health consequences of MAFLD, current diagnostic and management strategies remain inadequate. Liver biopsy remains the gold standard for diagnosing MAFLD, offering a direct assessment of hepatic steatosis and the ability to detect inflammation and fibrosis [11]. However, it has several limitations, including its invasive nature, sampling error, patient discomfort, and high cost, which render it less suitable for routine clinical use [12]. Therefore, there is a significant clinical need for a noninvasive, straightforward, and reliable predictor of fatty liver disease. Insulin resistance (IR), a key feature of metabolic syndrome, plays a pivotal role in the development of metabolic-related diseases, including fatty liver [13–15]. Identifying a reliable biomarker for IR could significantly improve early detection and intervention strategies related to metabolic disorders [16]. However, current methods for assessing IR are often complex and invasive, rendering them impractical for routine clinical application [17]. Consequently, there is a pressing need for noninvasive and straightforward methods to assess IR, which would enhance the clinical management of these conditions. The Triglyceride-Glucose (TyG) index and its derived indices offer a simple and feasible alternative for evaluating insulin resistance.

Recent studies have shown that the TyG index and its derived parameters are effective in predicting various disease states and tracking disease progression, especially in the assessment of NAFLD and the prediction of liver fibrosis [18,19]. In addition, the triglyceride glucose-body mass index (TyG-BMI), as an easily accessible and interpretable measurement indicator, has been further investigated in clinical research. However, the available evidence regarding the relationship between different TyG-BMIs and MAFLD is still limited. At present, the existing studies provide inadequate or no specific description of the relationship between TyG-BMIs and MAFLD, as well as different fatty liver phenotypes. This gap in knowledge significantly hampers the development of effective screening and intervention strategies for MAFLD. Therefore, this study aimed to explore the correlation between different TyG-BMIs and MAFLD, as well as different fatty liver phenotypes, to provide a more comprehensive understanding of the role of TyG-BMI in the diagnosis and management of MAFLD.

## Methods

### Study design and population

**Fig 1:**
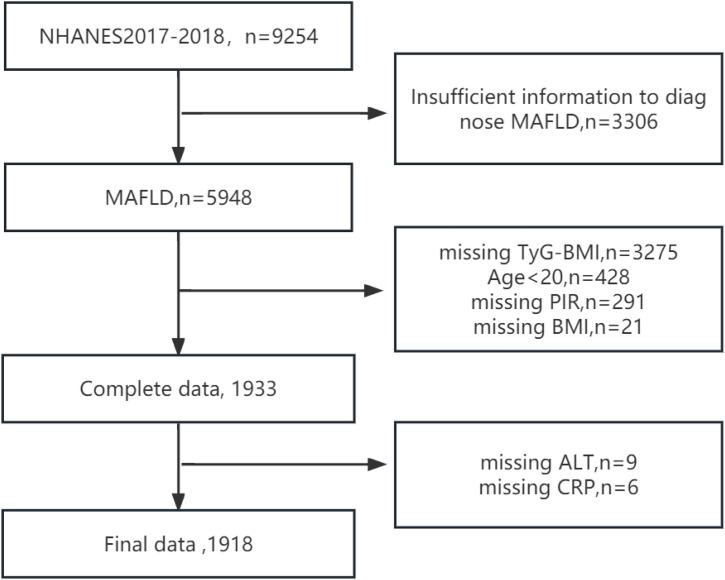
Flowchart and selection of study participants. MAFLD, metabolic-associated fatty liver disease; TyG-BMI, triglyceride–glucose–body mass index; PIR, ratio of income to poverty; BMI, body mass index; ALT, alanine aminotransferase; CRP, C-reactive protein.

This was a retrospective cross-sectional study using data from the 2017--2018 cycle of the National Health and Nutrition Examination Survey (NHANES). This cycle offers detailed vibration-controlled transient elastography (VCTE) data, along with an array of health-related measurements, physical examinations, and laboratory results. This database uses a complex multistage sampling design; thus, it can represent the characteristics of the entire U.S. population (https://www.cdc.gov/nchs/nhanes/about_nhanes.htm). This study was approved by the Ethics Review Committee of the National Health and Nutrition Examination Survey, and since all participants provided informed consent, our study did not require additional ethical review. During the 2017--2018 period, with a total of 9,254 participants, we employed the following recruitment process. Initially, we excluded 3,306 participants due to incomplete information, which prevented a definitive diagnosis of MAFLD. Additionally, we excluded 3,275 participants for whom the TyG-BMI could not be calculated. We also excluded participants younger than 20 years. We subsequently deleted missing data sequentially for the following variables: family income to poverty ratio (PIR), BMI, alanine aminotransferase (ALT), and C-reactive protein (CRP). Ultimately, the study included a total of 1,918 participants (Fig 1).

### Population statistics and laboratory measurement parameters

The variables included in our analysis included age, sex, race, marital status, education level, household income, daily activity level, BMI, waist circumference, and smoking status. We also assessed various metabolic-related conditions, including diabetes, coronary heart disease (CHD), and hypertension. All Population statistics parameters are taken at the mobile examination center (MEC). The laboratory test results included CRP, fasting blood glucose, serum albumin, ALT, aspartate aminotransferase (AST), gamma-glutamyltransferase (γ-GGT), triglyceride (TG), and uric acid (UA) levels. The laboratory test are available for sample persons fasting at least 8 hours or more but less than 24 hours.

### Measurement and calculation of TyG-BMI

To address the need for a noninvasive and reliable predictor of insulin resistance and its association with MAFLD, we calculated the TyG-BMI index as the independent variable in this study. The TyG-BMI index is derived from the TyG index, which is a widely used measure of insulin resistance. The TyG index is calculated using the following formula:


TyG=ln[fasting triglyceride(mg/dL)×fasting glucose(mg/dL)/2]

In this study, we extended the TyG index by incorporating BMI (Body Mass Index) to create the TyG-BMI index, which is calculated using the following formula:


$$\text{TyG}-\text{BMI}=\text{TyG Index}\times \text{BMI}(\text{kg}/{\text{m}}^{\text{2}})$$

### Definition of MAFLD without fibrosis and with fibrosis

We used the NHANES data from 2017 to 2018 because this cycle contains detailed VCTE examination data. The criterion for identifying hepatic fat deposition was a median controlled attenuation parameter (CAP) of 274 dB/m or higher, whereas the presence of liver fibrosis was indicated by a median liver stiffness measurement (LSM) of 6.3 kPa or higher [19]. In addition to the ultrasonographic identification of fatty liver, the diagnosis of MAFLD also requires the presence of at least one of the following metabolic condition: type 2 diabetes (T_2_DM), obesity, or evidence of metabolic dysregulation [20]. Metabolic dysfunction is defined as the presence of at least two metabolic risk abnormalities [21]. These abnormalities including a waist circumference of at least 102/88 cm for Caucasian men and women or at least 90/80 cm for Asian men and women; blood pressure of at least 130/85 mmHg or the use of antihypertensive medication; plasma triglycerides (TG) of at least 150 mg/dl or the use of triglyceride-lowering medication; plasma levels of high-density lipoprotein cholesterol (HDL-C) < 40 mg/dl in males and < 50 mg/dl in females, or if on medication to decrease cholesterol; prediabetes is characterized by plasma glucose levels between 100 and 125 mg/dl when fasting, 140 and 199 mg/dl after 2 hours postload, or 5.7% to 6.4% in glycosylated hemoglobin (HbA1c); a homeostasis model assessment (HOMA) score of greater than 2.5 indicates insulin resistance (IR); and high-sensitivity CRP levels greater than 2 mg/L.

### Statistical analysis

Normally distributed variables are reported as the mean ± standard deviation (SD), whereas variables with skewness are reported as the median (interquartile range [IQR]). The frequency and percentage (%) representations of categorical variables were used. To evaluate differences between the various phenotypes of MAFLD groups, statistical analysis was carried out via the chi-square test for categorical variables, one-way ANOVA for variables with a normal distribution, and the Kruskal‒Wallis H test for variables with a skewed distribution.

Threshold effect analysis was performed to evaluate the predictive power of TyG-BMI for MAFLD, and a limited cubic spline curve was fitted to the association between TyG-BMI and MAFLD incidence. Additionally, the connection between various phenotypes of fatty liver and TyG-BMI levels was modeled via ordered multinomial logistic regression. The different phenotypes of fatty liver considered in this study include: without MAFLD, MAFLD without fibrosis and MAFLD with fibrosis. Three groups were created from the research population according to varying TyG-BMI values (TyG-BMI < 143.82, 143.82 < TyG-BMI < 183.95, TyG-BMI > 183.95). After the independent variable was multiplied by 0.01, single-factor and multifactor logistic regression were employed to examine the relationships between various TyG-BMI values and distinct MAFLD phenotypes. The model’s stability was ascertained by varying various variables across several models. Gender and age were taken into account in Model 1. Additional modifications were made to Model 2 for BMI, education level, PIR, and physical activity. Model 3 required further modifications to CRP and fasting blood glucose levels. Finally, Model 4 included modifications for metabolic conditions such as hypertension, type 2 diabetes, coronary heart disease, and stroke. The 95% CIs and odds ratios (ORs) were used to express the results. To evaluate the relationships between VCTE parameters and liver function markers and the TyG-BMI, Pearson correlation was used.

WindFree Statistics version 1.9 and R 4.2.2 statistical tools were used for all studies. Notable differences are indicated with a p value less than 0.05.

## Results

### Baseline characteristics of the study population

A total of 1918 patients were included in the study, with an average age of 50.7 ± 17.3 years. The distribution characteristics of the different fatty liver phenotypes in the study population are presented in Table 1. The overall prevalence of MAFLD without fibrosis in the study population was 28.8%, and that of MAFLD with fibrosis was 16.1%. Significant differences were observed among the groups in terms of sex, age, race, marital status, activity, BMI, waist circumference, diabetes status, hypertension status, cotinine, CAP, SWE, CRP, glucose, TyG-BMI, albumin, UA, TG, ALT, AST, and γ-GGT (all *P* < 0.05). However, there were no significant differences in the distributions of patient characteristics such as education, PIR, smoking status and CHD among the different phenotypes of MAFLD groups (all *p* > 0.05).

**Table 1:** Baseline characteristics of participants with different MAFLD phenotypes.

Variables	Total (n = 1918)	Non-MAFLD (n = 1057)	MAFLD without fibrosis (n = 552)	MAFLD with fibrosis (n = 309)	*P*
Age(years)	50.7 ± 17.3	48.5 ± 18.1	52.8 ± 16.2	54.5 ± 15.2	< 0.001
Gender, n (%)					0.01
Male	945 (49.3)	488 (46.2)	290 (52.5)	167 (54)	
Female	973 (50.7)	569 (53.8)	262 (47.5)	142 (46)	
Race, n (%)					< 0.001
Mexican American	265 (13.8)	107 (10.1)	98 (17.8)	60 (19.4)	
Non-Hispanic White	677 (35.3)	353 (33.4)	203 (36.8)	121 (39.2)	
Non-Hispanic Black	436 (22.7)	277 (26.2)	99 (17.9)	60 (19.4)	
Other Hispanic	540 (28.2)	320 (30.3)	152 (27.5)	68 (22)	
Marry, n (%)					< 0.001
No	1140 (59.4)	578 (54.7)	366 (66.3)	196 (63.4)	
Yes	778 (40.6)	479 (45.3)	186 (33.7)	113 (36.6)	
Education level, n (%)					0.115
<9	136 (7.1)	70 (6.6)	40 (7.2)	26 (8.4)	
9-12	684 (35.7)	357 (33.8)	219 (39.7)	108 (35)	
>12	1098 (57.2)	630 (59.6)	293 (53.1)	175 (56.6)	
PIR, n (%)					0.311
≤1.3	531 (27.7)	298 (28.2)	152 (27.5)	81 (26.2)	
>1.3 and ≤3.5	803 (41.9)	431 (40.8)	226 (40.9)	146 (47.2)	
>3.5	584 (30.4)	328 (31)	174 (31.5)	82 (26.5)	
activity, n (%)					< 0.001
No	1453 (75.8)	755 (71.4)	443 (80.3)	255 (82.5)	
Yes	465 (24.2)	302 (28.6)	109 (19.7)	54 (17.5)	
BMI, Mean ± SD	29.8 ± 7.4	26.9 ± 5.8	31.6 ± 6.0	36.3 ± 9.2	< 0.001
Waist, Mean ± SD	100.5 ± 17.1	92.9 ± 14.2	106.1 ± 13.3	116.4 ± 17.7	< 0.001
Smoking status, n (%)					0.125
No	1083 (75.6)	615 (74.9)	319 (79)	149 (72)	
Yes	349 (24.4)	206 (25.1)	85 (21)	58 (28)	
CHD, n (%)					0.052
No	1828 (95.7)	1019 (96.7)	517 (94.3)	292 (94.5)	
Yes	83 (4.3)	35 (3.3)	31 (5.7)	17 (5.5)	
T_2_DM, n (%)					< 0.001
No	1603 (83.6)	964 (91.3)	449 (81.3)	190 (61.5)	
Yes	314 (16.4)	92 (8.7)	103 (18.7)	119 (38.5)	
Hypertension, n (%)					< 0.001
No	857 (45.6)	569 (55.2)	207 (38)	81 (26.6)	
Yes	1023 (54.4)	461 (44.8)	338 (62)	224 (73.4)	
CAP(dB/m)	266.6 ± 63.5	219.9 ± 37.1	316.3 ± 32.8	337.9 ± 37.7	< 0.001
SWE, (kPa)	5.0 (4.1, 6.2)	4.6 (3.8, 5.6)	4.8 (4.1, 5.5)	8.0 (6.8, 11.0)	< 0.001
CRP, (mg/L)	1.9 (0.8, 4.3)	1.4 (0.7, 3.2)	2.5 (1.2, 4.7)	3.5 (1.6, 6.7)	< 0.001
Glucose, (mmol/L)	6.4 ± 2.1	5.9 ± 1.6	6.6 ± 2.0	7.6 ± 3.0	< 0.001
TyG-BMI, n (%)					< 0.001
<143.82	639 (33.3)	556 (52.6)	66 (12)	17 (5.5)	
>143.82 and <183.95,	639 (33.3)	337 (31.9)	233 (42.2)	69 (22.3)	
>183.95	640 (33.4)	164 (15.5)	253 (45.8)	223 (72.2)	
Albumin, (g/L)	40.1 ± 3.2	40.4 ± 3.3	40.0 ± 3.1	39.5 ± 3.3	< 0.001
UA, (umol/L)	329.7 ± 88.0	312.1 ± 83.4	344.5 ± 84.5	363.3 ± 95.1	< 0.001
TG, Median (IQR)	94.0 (62.0, 137.8)	77.0 (54.0, 112.0)	113.0 (79.8, 159.0)	116.0 (82.0, 166.0)	< 0.001
ALT, (U/L)	18.0 (13.0, 26.0)	16.0 (12.0, 22.0)	20.0 (14.0, 28.0)	24.0 (17.0, 40.0)	< 0.001
AST, (U/L)	19.0 (16.0, 24.0)	19.0 (16.0, 23.0)	19.0 (16.0, 24.0)	22.0 (17.0, 30.0)	< 0.001
γ-GGT, (IU/L)	21.0 (15.0, 33.0)	18.0 (13.0, 27.0)	23.5 (17.0, 33.2)	30.0 (21.0, 54.0)	< 0.001

Note: PIR, ratio of income to poverty; BMI, body mass index; CHD, coronary heart disease; T_2_DM, type 2 diabetes; CAP, controlled attenuation parameter; SWE, shear wave elasticity; CRP, C-reactive protein; GLU, glucose; TyG-BMI, triglyceride–glucose–body mass index; UA, uric acid; TG, triglyceride; ALT, alanine aminotransferase; AST, aspartate aminotransferase; γ-GGT, gamma-glutamyltransferase.

### Nonlinear relationships between TyG-BMI and different phenotypes in MAFLD patients

**Fig 2:**
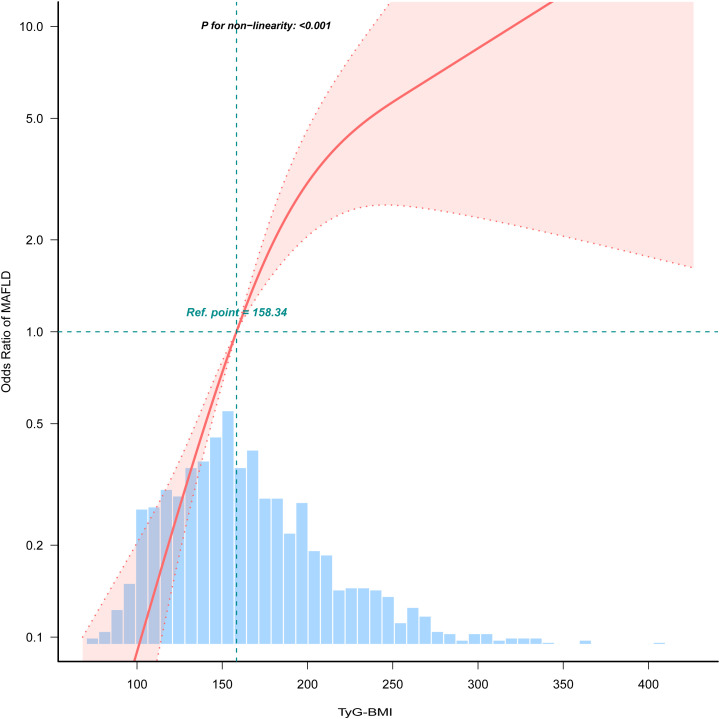
Limited cubic spline curve of the combined TYG-BMI index and the incidence of MAFLD. The red solid line represents the estimated odds ratio of MAFLD as a function of TyG-BMI, while the red dashed line indicates the 95% confidence interval. The green dashed line marks the reference point at TyG-BMI = 158.34. The vertical green dashed line represents the median point of the TyG-BMI distribution. The horizontal green dashed line signifies the null effect line. The statistical significance of non-linearity is denoted by *P* < 0.001, suggesting a significant non-linear relationship between TyG-BMI and MAFLD. MAFLD, metabolic-associated fatty liver disease; TyG-BMI, triglyceride–glucose–body mass index.

**Fig 3:**
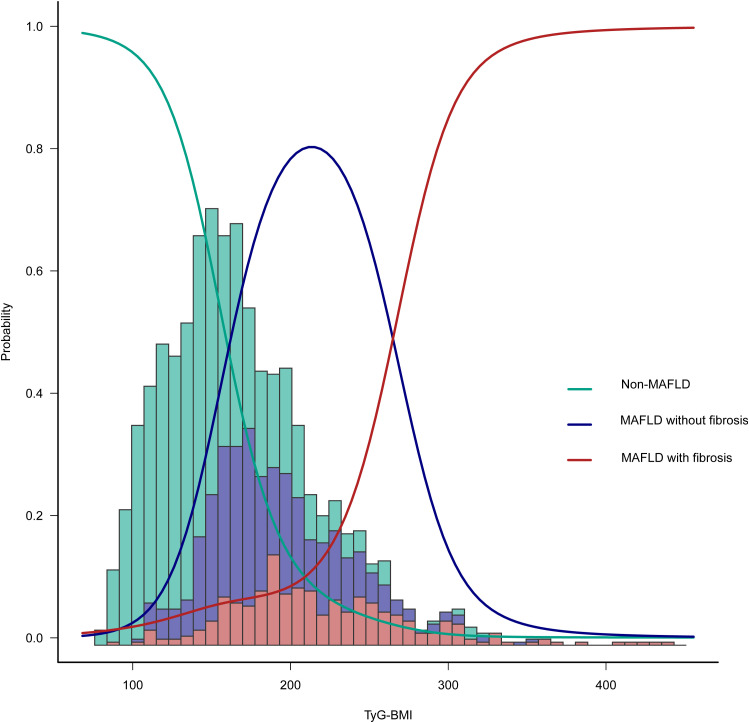
Limited cubic spline curve of the combined TyG-BMI and different fatty liver phenotypes. 1: non-MAFLD; 2: MAFLD without fibrosis; 3: MAFLD with fibrosis.

Multivariate logistic regression analysis revealed a nonlinear association between the TyG-BMI and the incidence of MAFLD (Fig 2). We employed a piecewise model to characterize the curvilinear relationship, identifying a critical threshold at a TyG-BMI of 176.783. On the left of this inflection point, the odds ratio for the occurrence of MAFLD was 1.044 (OR: 1.044, 95%CI: 1.029–1.059, *p* < 0.001). On the right of this inflection point, the OR for the occurrence of MAFLD was 1.016 (OR: 1.016, 95%CI: 1.003–1.029, *p* = 0.0127) (Table 2). Subsequent ordinal multinomial logistic regression, adjusted for several covariates, confirmed a similar curvilinear relationship between TyG-BMI and the various MAFLD phenotypes (Fig 3).

**Table 2:** Threshold effect analysis of the TyG-BMI on the incidence of MAFLD.

Item	Breakpoint.OR (95%CI)	*P* value
E_BK1	176.783 (174.941,178.625)	NA_character_
slope1	1.044 (1.029 ~ 1.059)	<0.001
slope2	1.016 (1.003 ~ 1.029)	0.0127

### Multivariate regression analysis of TyG-BMI and different MAFLD phenotypes

When TyG-BMI underwent a 0.01-fold conversion, in the univariate ordinal multinomial logistic regression analyses, TyG-BMI, treated as a continuous variable with each unit increase, demonstrated a positive association with the likelihood of developing various MAFLD phenotypes. Specifically, for MAFLD without fibrosis, the OR was 11.29 (95% CI: 8.38–15.21, *p* < 0.001), and for MAFLD with fibrosis, the OR was 37.24 (95% CI: 25.92–53.51, *p* < 0.001). TyG-BMI levels were divided into three groups, After adjusting for potential confounders, as detailed in Table 3, Model 5 revealed that the relationship, although somewhat reduced, remained statistically significant. A higher TyG-BMI was significantly associated with an increased probability of MAFLD without fibrosis (OR = 5.99, 95% CI = 3.25–11.06; *p* < 0.001). Similarly, the probability of developing MAFLD with fibrosis also increased significantly (OR = 5.24, 95% CI = 2.28–12.06, *p* < 0.001).

**Table 3:** Multivariate regression analysis of TyG-BMI with different phenotypes of MAFLD.

Variable	MAFLD without Fibrosis	*P* value	MAFLD with Fibrosis_	*P* value
	OR (95% CI)		OR (95% CI)	
TyG.BMI	11.29 (8.38 ~ 15.21)	<0.001	37.24 (25.92 ~ 53.51)	<0.001
Model 1				
T1	Ref		Ref	
T2	5.83 (4.3 ~ 7.91)	<0.001	6.71 (3.88 ~ 11.61)	<0.001
T3	13.01 (9.43 ~ 17.96)	<0.001	44.57 (26.41 ~ 75.23)	<0.001
Model 2				
T1	Ref		Ref	
T2	5.44 (4 ~ 7.39)	<0.001	6.01 (3.46 ~ 10.41)	<0.001
T3	13.04 (9.43 ~ 18.03)	<0.001	45.04 (26.6 ~ 76.26)	<0.001
Model 3				
T1	Ref		Ref	
T2	4.73 (3.16 ~ 7.1)	<0.001	2.69 (1.37 ~ 5.28)	0.004
T3	7.46 (4.16 ~ 13.38)	<0.001	7.72 (3.48 ~ 17.09)	<0.001
Model 4				
T1	Ref		Ref	
T2	4.43 (2.94 ~ 6.67)	<0.001	2.44 (1.23 ~ 4.82)	0.011
T3	6.35 (3.47 ~ 11.62)	<0.001	5.81 (2.55 ~ 13.23)	<0.001
Model 5				
T1	Ref		Ref	
T2	4.38 (2.89 ~ 6.63)	<0.001	2.33 (1.17 ~ 4.64)	0.016
T3	5.99 (3.25 ~ 11.06)	<0.001	5.24 (2.28 ~ 12.06)	<0.001

The multivariate linear regression model was adjusted for covariates, and the variable was reduced by a factor of 100 before a multiple regression analysis was conducted. T1: TyG-BMI < 143.82, T2: 143.82 < TyG-BMI < 183.95, T3: TyG-BMI > 183.95.

Model 1: unadjusted,

Model 2: Age, sex,

Model 3: Model 2 + BMI, education, PIR, activity, race, and smoking status,

Model 4: Model 3 + CRP, glucose,

Model 5: Model 4 + CHD, T_2_DM, Stroke, Hypertension.

### Correlation analysis of the TyG-BMI and VCTE

**Fig 4:**
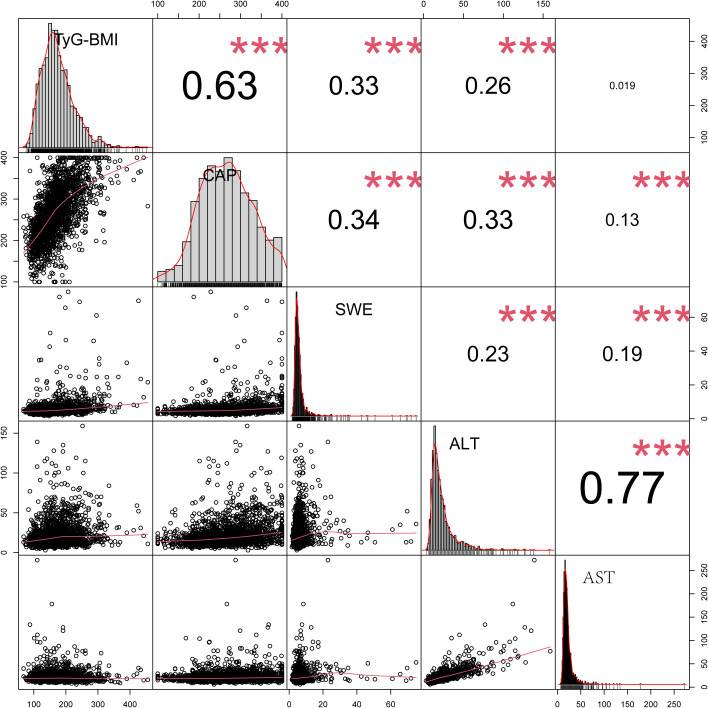
Pearson correlation coefficients for the TyG-BMI in the adult population (n = 1918): NHANES 2017–2018. TyG-BMI, triglyceride–glucose–body mass index; CAP, controlled attenuation parameter; SWE, shear wave elasticity; ALT, alanine aminotransferase; AST, aspartate aminotransferase.

As depicted in Fig 4, significant correlations were observed between TyG-BMI and CAP, SWE, and ALT (all p values < 0.05). The correlation coefficients, along with their 95% confidence intervals, are as follows: TyG-BMI with CAP: 0.60 (95% CI = 0.57–0.63), TyG with SWE: 0.29 (95% CI = 0.25–0.33), and TyG-BMI with ALT: 0.20 (95% CI = 0.16–0.25).

## Discussion

A substantial nonlinear connection between TyG-BMI and the incidence of MAFLD was discovered in this cross-sectional study of adult Americans. The threshold value of TyG-BMI = 176.78 that we identified marks a transition point where the risk of MAFLD shifts from a sharp increase to a more gradual rise. Clinically, this threshold can assist physicians in risk stratification for MAFLD, to determine whether more aggressive management measures or closer monitoring are necessary. Therefore, incorporating the TyG-BMI threshold as part of a comprehensive risk assessment tool can aid in the development of public health policies and preventive measures. Compared with the non-MAFLD group, the probability of various MAFLD phenotypes (MAFLD without fibrosis, OR: 11.29, 95% CI: 8.38–15.21; MAFLD with fibrosis, OR: 37.24, 95% CI: 25.92–53.51) was positively correlated with TyG-BMI when viewed as a continuous variable. Furthermore, when the TyG-BMI was used as a categorical variable, even after adjusting for age, sex, BMI, education status, PIR, activity status, race, smoking status, CRP, glucose, CHD, T_2_DM, stroke status and hypertension, this relationship remained statistically significant, despite slight attenuation (MAFLD without fibrosis, OR: 5.99, 95% CI: 3.25–11.06; MAFLD with fibrosis, OR: 5.24, 95% CI: 2.28–12.06). The strong positive correlation between the TyG-BMI and CAP and SWE supports the adoption of the TyG-BMI as an alternative screening instrument for MAFLD. Future studies need to further evaluate the correlation between TyG-BMI and the occurrence of MAFLD under diverse CAPs, as well as SWE, to determine the robustness of the study results [21].

Males with obesity, diabetes, hypertension, dyslipidemia, metabolic syndrome, or increased ALT and AST levels had a significantly greater frequency of all grades of steatosis and fibrosis, according to a nationwide study conducted in China [22]. Similarly, we also found that males, diabetic patients, and those with low activity were more inclined to have severe MAFLD phenotypes, as shown in Table 1. In a study aimed at NAFLD and physical activity, moderate amounts of physical activity were associated with lower odds of NAFLD, and the highest levels of physical activity were associated with significantly lower odds of advanced fibrosis. In addition, the levels of CAP, SWE, UA, glucose, TG, ALT, AST and γ-GGT increased with increasing MAFLD phenotype. These results reflected the metabolic dysfunction (such as dyslipidemia, abnormal liver function, and glucose metabolism disorders) of MAFLD. Several studies have demonstrated that TyG-related parameters have potential for predicting fatty liver and liver fibrosis [18,19,23–26]. Mingxing Chang et al. reported that individuals with higher TyG-related indices were more likely to have MAFLD [27]. In particular, the TyG-BMI exhibited the strongest predictive performance among the indices, the area under the curve (AUC) for diagnosing MAFLD was greater than 0.8 in multiple investigations [26,28–30], and participants in the highest TyG-BMI quartile group were 380.87 times more likely to have MAFLD than those in the lowest quartile group [27]. Most other studies have also shown that the TyG-BMI is more suitable for early screening of NAFLD and MAFLD [19,24,26]. The potential biological mechanisms may include adipose tissue dysfunction and IR. Higher BMI, is associated with adipose tissue dysfunction characterized. Dysregulated metabolic mediators released from adipose tissue, including cytokines, adiponectin, chemokines, excessive lipids, and toxic lipid metabolites, promote insulin resistance (IR) in other tissues and systemic inflammation [13]. Research findings indicated that there is a significant epidemiological association between IR and MAFLD [31] and that this association can be non-invasively assessed using the TyG index [19,32]. The biomarker TyG-BMI selected in our study integrates TG, FPG, and BMI to assess IR. Elevated levels of TyG-BMI indicate significant IR, which leads to reduced hepatic insulin sensitivity [23]. This, in turn, promotes lipolysis and the release of free fatty acids (FFAs) from adipose tissue. These FFAs accumulate in the liver, contributing to hepatic steatosis and the progression of MAFLD [31].

Nevertheless, the majority of these studies have not offered a thorough examination of the link between the TyG-BMI and MAFLD, simply assessing the efficacy of the TyG-BMI in predicting MAFLD. Furthermore, the associations between different TyG-BMIs and various phenotypes of fatty liver, as well as the inflection point of the nonlinear relationship between these levels and the prevalence of MAFLD, have not been fully investigated in many studies. The study of Rong et al*.* described a dose–response relationship between the two factors [25]. Our study revealed a significant positive correlation between TyG-BMI and the incidence of MAFLD, with a limited cubic spline curve shown as a reverse J-shaped relationship between TyG-BMI and MAFLD, and identified a turning point at TyG-BMI = 176.783. In addition, for different MAFLD phenotypes, a similar nonlinear relationship was observed. With increasing TyG-BMI, the likelihood of having no MAFLD gradually decreased, whereas the incidence of MAFLD without fibrosis initially tended to increase but then decreased. Furthermore, the incidence of MAFLD with fibrosis significantly increased with increasing TyG-BMI.

The study findings were based on high-quality anthropometric and laboratory data from the NHANES database, which is comprehensive and representative of the population at the national level. Furthermore, there are few related clinical studies on MAFLD, so our findings contribute some evidence to the scant research. Finally, the indices evaluated in this study rely on routine biochemistry parameters that do not require rare, difficult-to-obtain, or expensive tests. Nevertheless, our study also has several limitations. First, owing to the cross-sectional nature of the study, we were unable to establish a temporal association between the TyG-BMI and MAFLD, which restricts our ability to establish a causal relationship between TyG-BMI and MAFLD. Future longitudinal studies should aim to track changes in TyG-BMI over time and assess their long-term effects on liver health and metabolic outcomes. Second, we analyzed data from only a single cycle year of the NHANES database, and our results were based on a sample of the general population without weighting the data, which may not be representative of the entire U.S. population. To examine the relationships between TyG-BMI and the incidence of MAFLD and its many manifestations, we intend to concentrate on Chinese populations in our future research and include longitudinal data on trajectory changes in the TyG-BMI index. Third, even with regression models and sensitivity analyses, residual confounding effects from unmeasured or unknown factors cannot be completely ruled out. Fourth, although VCTE is an extremely sensitive test for liver fibrosis and steatosis, its specificity may be compromised, thereby underestimating the prevalence of both fibrosis and MAFLD.

## Conclusion

This study aimed to investigate the associations between TyG-BMI and MAFLD, along with its various phenotypes. After adjusting for confounding variables, a nonlinear relationship between TyG-BMI and MAFLD, as well as its different phenotypes, was observed. The indices assessed in this study are based on routine biochemistry parameters and do not require rare, difficult-to-obtain, or expensive tests. Their calculation is straightforward and simple, facilitating their easy application in clinical settings to predict the presence of MAFLD.
